# Fast Healthcare Interoperability Resources, Clinical Quality Language, and Systematized Nomenclature of Medicine—Clinical Terms in Representing Clinical Evidence Logic Statements for the Use of Imaging Procedures: Descriptive Study

**DOI:** 10.2196/13590

**Published:** 2019-05-13

**Authors:** Eseosa Odigie, Ronilda Lacson, Ali Raja, David Osterbur, Ivan Ip, Louise Schneider, Ramin Khorasani

**Affiliations:** 1 Center for Evidence-Based Imaging Brigham and Women's Hospital Brookline, MA United States; 2 Harvard Medical School Boston, MA United States; 3 Countway Medical Library Harvard Medical School Boston, MA United States

**Keywords:** knowledge representation, guidelines, evidence-based medicine, clinical decision support

## Abstract

**Background:**

Evidence-based guidelines and recommendations can be transformed into “If-Then” Clinical Evidence Logic Statements (CELS). Imaging-related CELS were represented in standardized formats in the Harvard Medical School Library of Evidence (HLE).

**Objective:**

We aimed to (1) describe the representation of CELS using established Systematized Nomenclature of Medicine—Clinical Terms (SNOMED CT), Clinical Quality Language (CQL), and Fast Healthcare Interoperability Resources (FHIR) standards and (2) assess the limitations of using these standards to represent imaging-related CELS.

**Methods:**

This study was exempt from review by the Institutional Review Board as it involved no human subjects. Imaging-related clinical recommendations were extracted from evidence sources and translated into CELS. The clinical terminologies of CELS were represented using SNOMED CT and the condition-action logic was represented in CQL and FHIR. Numbers of fully and partially represented CELS were tallied.

**Results:**

A total of 765 CELS were represented in the HLE as of December 2018. We were able to fully represent 137 of 765 (17.9%) CELS using SNOMED CT, CQL, and FHIR. We were able to represent terms using SNOMED CT in the temporal component for action (“Then”) statements in CQL and FHIR in 755 of 765 (98.7%) CELS.

**Conclusions:**

CELS were represented as shareable clinical decision support (CDS) knowledge artifacts using existing standards—SNOMED CT, FHIR, and CQL—to promote and accelerate adoption of evidence-based practice. Limitations to standardization persist, which could be minimized with an add-on set of standard terms and value sets and by adding time frames to the CQL framework.

## Introduction

### Background

Imaging clinical decision support (CDS) applies health information technology (IT) to inform clinical decision making at the point of care regarding the need for imaging or the optimal study based on the best available evidence [1]. Legislation has called for the use of health IT, including CDS, for health promotion and health quality improvement [2,3]. Subsequently, regulations promulgated in response to the Protecting Access to Medicare Act (PAMA) state that health care providers should reference appropriate use criteria or evidence-based clinical knowledge while ordering certain advanced imaging exams [4]. Such an evidence-based approach to appropriate medical imaging by way of CDS systems can help mitigate health care costs and imaging utilization, while providing appropriate and safe health care to those who require these procedures [5-7].

Many guidelines, recommendations, systematic reviews, and clinical decision rules have been published or endorsed by national societies in the peer-reviewed literature and as best practices by other provider groups related to appropriate use of advanced imaging procedures for certain indications. The knowledge contained in these recommendations and guidelines can be transformed into Clinical Evidence Logic Statements (CELS) that can be implemented into CDS systems. However, to be widely shared and usable in such systems, CELS must be translated into established standardized syntax and formats such as Systematized Nomenclature of Medicine—Clinical Terms (SNOMED CT) [8], Clinical Quality Language (CQL) [9], and Fast Healthcare Interoperability Resources (FHIR) [10]. SNOMED International does not charge for the use of SNOMED CT in SNOMED International Member countries or territories; CQL and FHIR are Health Level Seven (HL7) standards and are available at no cost under a licensing agreement by which HL7 will retain its copyright. Key components of each standard are summarized in the subsections that follow.

### Systematized Nomenclature of Medicine—Clinical Terms Compositional Grammar

SNOMED CT compositional grammar is a standard ontology for representing clinical concepts and establishes relationships between them [8]. Clinical terms such as “X-ray knee” can be modeled in SNOMED CT, where each concept is linked to an identifying number. Concepts in SNOMED CT are organized into expressions. Precoordinated expressions are represented by a single concept identifier. Postcoordinated expressions are those that are represented by combining two or more concept identifiers. SNOMED CT establishes rules and hierarchies that define attributes, qualifiers, and relationships between concepts [11]. SNOMED CT also enables reference sets, which can be used to group SNOMED CT components (ie, concepts).

### Health Level Seven Clinical Quality Language Standard

The HL7 CQL Specification was developed to standardize the representation of clinical logic for clinical quality improvement [12]. More specifically, CQL was developed with the target of harmonizing expression logic. An additional component of the CQL Standard is the Expression Logical Model (ELM) [12]. Each CQL logic file is also represented as an ELM Extensible Markup Language (XML) document, which allows for an action to be represented for CDS. CQL files can reference clinical terms represented using SNOMED CT [8]. CQL files can also reference data models, such as the Quality Information and Clinical Knowledge (QUICK) logical model [13]. The QUICK data model defines the format and structure of the “retrieve” expressions in a CQL library. The retrieve declaration gathers a list of clinical data that is specific to the context of the patient or the population and to the retrieve itself.

### Fast Healthcare Interoperability Resources

FHIR is a standard for sharing health care information with multiple functional areas known as resources [10]. These modules or individual components can be combined into a framework that can be implemented in a health care system. The modules are generated in a format that can be recognized and utilized by most health care systems, while also allowing for flexibility and customization of these resources through extensions. Data representation in FHIR can be in the XML, JavaScript Object Notation (JSON), or Turtle formats and it uses both CQL and SNOMED CT standards in its representations. The FHIR “decisionsupportrule” resource [14], expressed through the ELM, represents shareable knowledge artifacts for CDS.

The Harvard Medical School Library of Evidence (HLE) provides a repository of medical evidence, publicly available from the HLE website, from a range of recommendation sources that can be utilized in CDS systems [15,16]. Each unit of medical evidence is represented as a CELS of “If-Then” logic statement form (eg, If [age>X] And [symptom] Then Not [procedure]). We aimed to (1) describe the representation of CELS using the established standards of SNOMED CT, CQL, and FHIR and (2) assess the limitations of using these standards to represent the CELS in the HLE.

## Methods

### Study Design and Setting

This descriptive study was exempt from the requirement of review from the Institutional Review Board as it did not include human subjects. The HLE currently contains imaging-related recommendations from clinical decision rules, professional society guidelines, and locally developed best practice guidelines [17]. As of December 20, 2018, there were a total of 765 completely graded CELS from 134 evidence sources in the HLE. A total of 235 of the CELS are Choosing Wisely content [18], pertaining to Priority Clinical Areas (eg, cervical or neck pain and suspected pulmonary embolism) specified by the Centers for Medicare and Medicaid Services [4,19].

### Representing Clinical Evidence Logic Statements in Established Standards: An Overview

**Figure 1:**
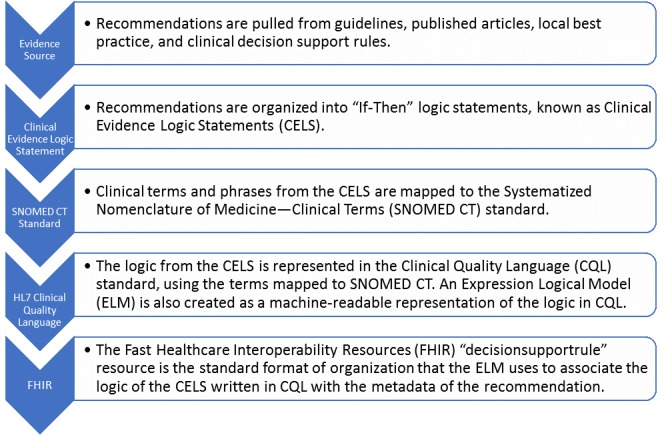
Relationship between the standards. HL7: Health Level Seven.

Steps in the process of translating a unit of evidence into FHIR so that it can be used in CDS are summarized in Figure 1. Recommendations are extracted from evidence sources, including published guidelines, recommendations, systematic reviews, clinical decision rules, and local best practices; each extracted recommendation is known as a unit of evidence. Each unit of evidence is then organized into an “If [condition] Then [action]” format which is known as a CELS. Therefore, each CELS consists of clinical terms and logic operators and has associated metadata (eg, source and author).

### Transforming a Unit of Evidence to Fast Healthcare Interoperability Resources: An Example

#### Overview

An in-depth transformation of a unit of evidence to FHIR is described below using a peer-reviewed article with recommendations for using ventilation-perfusion single-photon emission computed tomography (VQ SPECT) imaging for diagnosing pulmonary embolism [20]. The article recommends using VQ SPECT in patients with suspected pulmonary embolism (PE), and can be written as the following CELS: “If [Suspected PE] Then [VQ SPECT].”

Previous studies related to the HLE have identified three main types of variations in logic: single-decision statements, branching statements, and score-based statements [16]. The “Suspected PE” recommendation is an example of a single-decision statement.

#### Representing the Terms Using Systematized Nomenclature of Medicine: Clinical Terms

We modeled “Suspected PE” in SNOMED CT as follows: code “suspected PE”: '417113001'. This is an example of a precoordinated match.

#### Representing Clinical Logic in Clinical Quality Language

The CQL file is structured into a series of categories including the following:

1. Library: this is the name of the reference file, which is referenced by the secondary ELM file needed for each clinical decision.
2. Using: this term defines the data model that will be used (eg, QUICK).
3. Code System: this identifies the standardized code system, such as SNOMED CT.
4. Value Set: this identifies the specific codes within the code system that will be referenced in the clinical logic; either extensional or intensional value sets can be used.
5. Context: this can either be patient or population. For clinical logic in the HLE, the context is patient, as most data references the patient.
6. Define: this is a statement that creates a local name for conditions (eg, in an “If” statement).

**Figure 2:**
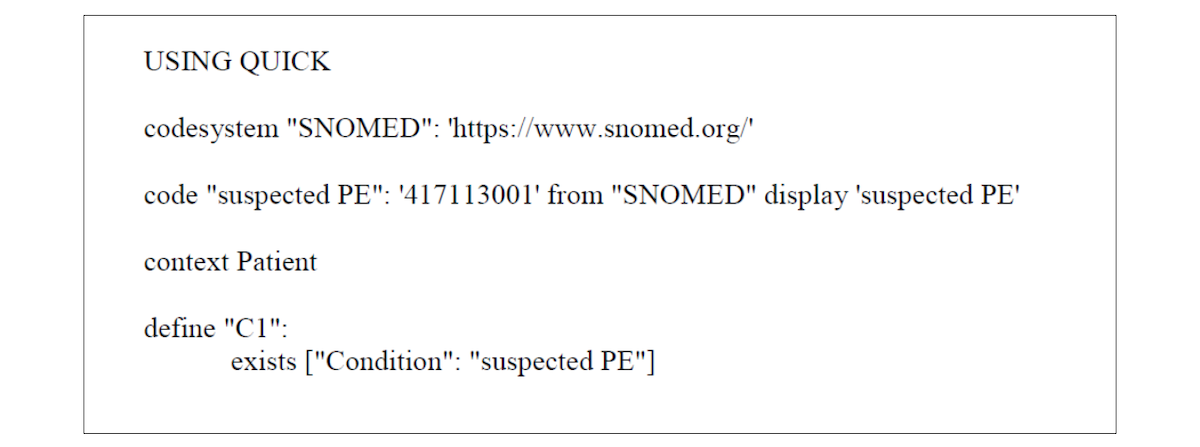
Suspected pulmonary embolism Clinical Quality Language (CQL) file. QUICK: Quality Information and Clinical Knowledge; SNOMED: Systematized Nomenclature of Medicine; PE: pulmonary embolism.

Although one can define an infinite number of subsets, these *define* statements should be organized and succinct. Naming the *define* statements creates a local name for all the conditions and rules that either exist or do not exist to make up a defined statement subset; this also allows one to reference the list of conditions in a future *define* statement, so that the *define* statements can be stacked. In most cases, the last *define* statement in the CQL file will be a *define* statement that contains all the conditions in the “If” statement that must be true to initiate the “Then” portion of the recommendation. A complete CQL file is shown in Figure 2.

#### Representation in Fast Healthcare Interoperability Resources

**Figure 3:**
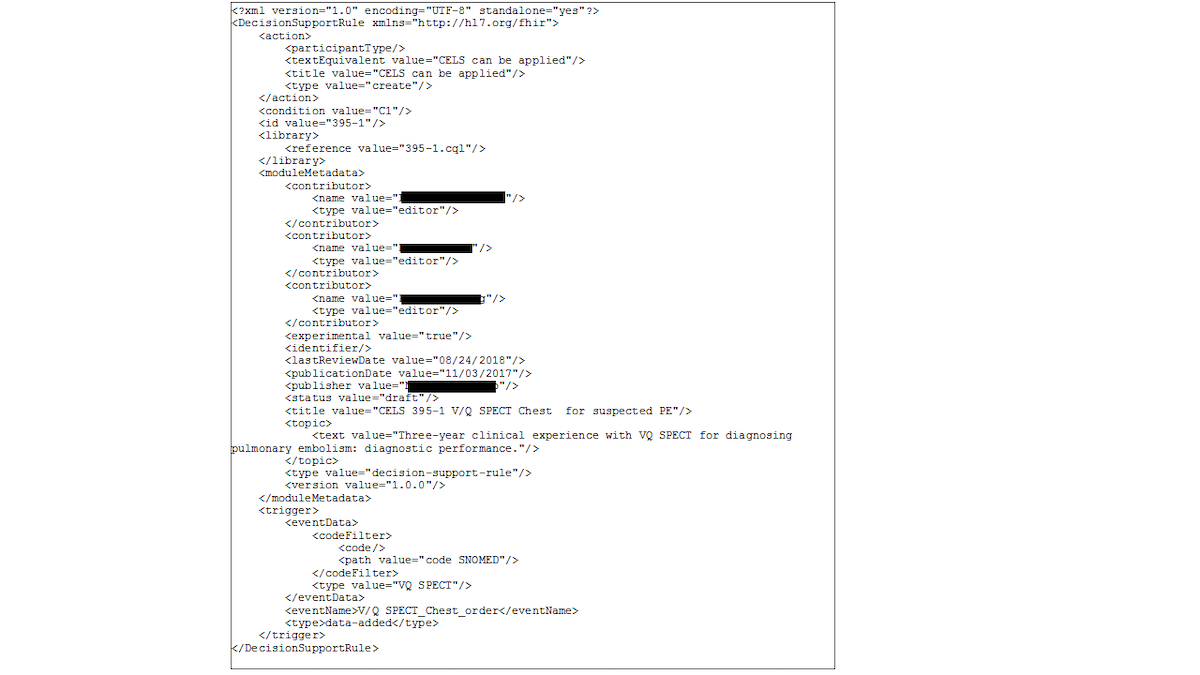
Suspected pulmonary embolism Extensible Markup Language (XML) file. HL7: Health Level Seven; FHIR: Fast Healthcare Interoperability Resources; CELS: Clinical Evidence Logic Statement; VQ SPECT: ventilation-perfusion single-photon emission computed tomography; PE: pulmonary embolism; SNOMED: Systematized Nomenclature of Medicine.

The ELM file (see Figure 3) is the second file necessary to share clinical logic written in CQL. As mentioned previously, the ELM is a machine-readable, canonical representation of the CQL logic, which is the intermediate step in implementing the logic written in CQL. This is where the Event, Context, and Actions are defined. The organization of the ELM file is dictated by the FHIR standard “decisionsupportrule” resource. It can be formatted in the XML, JSON, or Turtle formats; HLE uses XML.

The setup of the ELM file is shown in Textbox 1. Each individual decision rule, which thereby contains an individual action, has its own XML file.

**Setup of the Expression Logical Model (ELM) file.**

<action></action>: This portion of the Extensible Markup Language (XML) file references whether the clinical logic deems the test inappropriate or appropriate. The action can either be “Rule can be applied” if the test is appropriate or “Rule cannot be applied” if the test is not appropriate.

<condition value = />: In this part of the file, the name of the final *define* statement that contains all the conditions that renders the action true is referenced. Thus, a subset of clinical logic is referenced.

<moduleMetadata></moduleMetadata>: General information about the library file is referenced (eg, author names and title).

<library></library>: This portion of the XML file references the name of the Clinical Quality Language (CQL) file that contains the logic relevant to the action and condition.

<trigger></trigger>: This portion of the XML file references the clinical order that is related to the clinical logic and contains the event that triggers a decision rule. This trigger is defined in the implementation environment, and not defined in CQL.

The FHIR framework allows for the combination of multiple CQL files with their corresponding XML files. FHIR supports single-decision statements, branching-decision statements, and score-based statements.

### Representation of Branching Statements

Branching statements are recommendations that are applicable to patients with similar indications but fulfil various criteria; for example, recommendations for managing pulmonary embolism in pregnant patients versus nonpregnant patients. Thus, there are more than two CELS generated for the evidence source. The first step of translating these units of evidence is creating a decision tree. Each end point of the decision tree corresponds to a CELS, to be represented in CQL.

### Representation of Score-Based Statements

Score-based units of evidence also produce more than two CELS, corresponding to evidence-based scores; for example, recommendation for managing acute appendicitis for an Acute Inflammatory Response (AIR) score of 5. The ELM files created for each CQL file in the branched and score-based statement follow the same format as the ELM files in the single-decision statement.

### Assessing Representation of Clinical Evidence Logic Statements in Established Standards

The HLE contained a total of 2616 CELS at the time of data analysis for this publication. Among these, we counted the number of CELS that we were able to represent in SNOMED CT, CQL, and FHIR and reported this as a percentage of the total number of cells. For each of these, we characterized those CELS that could not be represented in SNOMED CT and those that could not be represented in CQL. CELS were defined as represented in SNOMED CT when all terms in the CELS could be represented using SNOMED CT. CELS are defined as represented in CQL when the action of the CELS, after the “Then” portion, could be represented in the ELM in the FHIR format.

## Results

We were able to represent terms using SNOMED CT in the temporal component for action (“Then”) statements in CQL and FHIR in 755 of 765 (98.7%) of CELS. Of the completely graded 765 CELS in the evidence library, 17.9% (n=137) were fully represented using SNOMED CT, CQL, and FHIR (see Table 1).

Reasons why CELS were not adequately represented are included in Textboxes 2-4 and are summarized as follows:

1. Clinical terms are unrepresented using SNOMED CT. Some clinical terms within logic statements contained one or more clinical terms not represented in SNOMED CT (eg, AIR score for acute appendicitis) [21-31].
2. Standard English phrases were unrepresented using SNOMED CT. Some common phrases that were not represented using SNOMED CT include “new feature” or “vehicle rollover.”
3. Temporal phrases were unrepresented in CQL. An additional number of CELS were not adequately represented as the “Then” portion of the logic statement because a temporal component could not be represented in CQL (eg, computed tomography [CT] chest in 12 months) and, subsequently, with the FHIR “decisionsupportrule” resource (see Textbox 4).

**Table 1:** Partially represented CELS^a^ in the Harvard Medical School Library of Evidence.

Type of CELS	Number of CELS (N=765), n (%)
CELS fully represented using SNOMED CT^b^, CQL^c^, and FHIR^d^	137 (17.9)
CELS partially represented using SNOMED CT	628 (82.1)
CELS partially represented due to CQL	10 (1.3)

^a^CELS: Clinical Evidence Logic Statement.

^b^SNOMED CT: Systematized Nomenclature of Medicine—Clinical Terms.

^c^CQL: Clinical Quality Language.

^d^FHIR: Fast Healthcare Interoperability Resources.

**Clinical terms unrepresented using Systematized Nomenclature of Medicine—Clinical Terms (SNOMED CT).**

- Acute Inflammatory Response (AIR) score [21]
- Alvarado score [22]
- Canadian Computed Tomography (CT) Head Rule [23]
- Canadian Cervical Spine Rule (CCSR) [24]
- New Orleans/Charity head trauma rule [25]
- National Emergency X-Radiography Utilization Study (NEXUS) head trauma rule [26]
- Magnetic resonance imaging (MRI) shoulder with dedicated metal suppression protocol
- O_2_ saturation on room air
- Optimizing imaging in suspected appendicitis (OPTIMAP) score [27]
- Revised Geneva (rGeneva) score [28]
- Simple calculated osteoporosis risk estimation (SCORE) score [29]
- Simplified Motor Score (SMS) [30]
- Sex, timing, origin, nausea, erythrocytes (STONE) score [31]

**Standard English phrases unrepresented using Systematized Nomenclature of Medicine—Clinical Terms (SNOMED CT).**

- New feature
- Suitable candidate
- Time-of-flight (TOF) magnetic resonance angiography (MRA)
- Vehicle rollover

**Temporal phrases unrepresented in Clinical Quality Language (CQL).**

- Computed tomography (CT) chest in 12 months
- CT chest in 18-24 months
- CT chest in 3 months, 9 months, And 24 months
- CT chest in 3-6 months
- CT chest in 6-12 months
- CT chest in 9-12 months And 24 months
- Low-dose CT annually for 3 years

Examples of partially unrepresented CELS include:

1. Alvarado score for suspected appendicitis; this is an example of an evidence source with three partially represented CELS, since the term “Alvarado score” does not exist in the SNOMED CT standard ontology, as indicated by the asterisk:
  - If [Alvarado score* >=4] And [Alvarado score* <=6] Then [CT abdomen]
  - If [Alvarado score* <4] Then Not [CT Abdomen]
  - If [Alvarado score* >6] Then Not [CT Abdomen]
2. Guidelines for management of small pulmonary nodules detected on CT scans—a statement from the Fleischner Society [32]:
  - If [pulmonary nodule on chest CT] And [nodule size <=4mm] And [high risk] Then [CT chest in 12 months]
  - If [pulmonary nodule on chest CT] And [nodule size >4mm] And [nodule size <=6] And [low risk] Then [CT Chest in 12 months]
  - If [pulmonary nodule on chest CT] And [nodule size >4mm] And [nodule size <=6] And [high risk] Then [CT chest in 6-12 months]

These CELS are examples of partially represented CELS due to actions such as “CT chest in 6-12 months” and “CT chest in 12 months.” These actions contain a future temporal component that cannot be represented in CQL. CT chest can be represented in a define statement. However, a define statement in CQL for scheduling a procedure at a future time cannot be created. Furthermore, value sets for terms such as “high risk” are not available.

## Discussion

### Principal Findings

Overall, 17.9% (137/765) of CELS were represented as shareable CDS knowledge artifacts using existing standards, SNOMED CT, FHIR, and CQL to promote and accelerate adoption of evidence-based practice. More work to represent imaging-related CELS need to be undertaken to standardize clinical knowledge included in the HLE. A few limitations to utilizing these standards for CDS implementation in the evidence library were identified. While SNOMED CT is robust, some terms do not exist in its ontology. For example, names for known rules or scores such as “Revised Geneva score” [28] cannot be represented. The HLE is currently in the process of creating an add-on set of terms in SNOMED CT so that these terms will have an ID and mapping.

In addition, English words, which contribute to the meaning of a clinical recommendation (eg, “high risk” and “suitable candidate”), may not be represented using SNOMED CT. In those situations, one can substitute terms that are in SNOMED CT that are a synonym or close in meaning to the original term. Use of value sets to enumerate concepts that may map to a criterion in a recommendation may be useful. However, these mappings are not always exact and may change the interpretation of the clinical recommendation. This limitation can also possibly be amended through an add-on set of terms to SNOMED CT. SNOMED CT is updated twice yearly and updates can include newly added concepts. In addition, developing an add-on set of terms can be expedited by creating more value sets, specifically sets of code from hierarchy-based definitions that are algorithmically defined (ie, intensional value sets) or enumerated (ie, extensional value sets). These can be disseminated publicly (eg, via the Value Set Authority Center) to accelerate cross-organizational efforts for terminology standardization. More importantly, guidelines and recommendations should be limited to using standardized terminology prior to getting published.

The limitations of the FHIR framework include determining ways to represent temporal actions and phrases. CQL gives rise to a temporal framework as the time frame of a condition can be defined; for example, “If CT chest in the past 12 months” can be represented. However, this allowance is limited to conditions within the “If” statement, and currently there is no temporal framework in the CQL file or future temporal component in the ELM file that runs an action (eg, “CT chest in 12 months” cannot be written in the XML file). Further, recurring actions such as “9-12 months And 24 months” cannot be represented. The current actions determine whether imaging at that exact time is appropriate or not. Clinical logic could be restructured in the “If-Then” statement to incorporate the time frame into the “If,” but this does not work in every situation. A systemized and structured approach to these statements with time frames should be added onto the CQL and FHIR framework by developers or CDS implementation services that expands upon the representation of time within the actions of CDS.

To summarize, a unit of evidence in the HLE is structured as a CELS. We represented terms using a standard terminology, SNOMED CT. The conditions or “If” statements are represented in CQL. Using the FHIR resource “decisionsupportrule,” we combine the action and the condition to represent CDS knowledge artifacts. CELS are publicly available and represented using existing standards to promote and accelerate adoption of evidence into daily practice to improve the quality of care and reduce waste.

### Conclusions

CELS were represented as shareable CDS knowledge artifacts using existing standards—SNOMED CT, FHIR, and CQL—to promote and accelerate adoption of evidence-based practice. However, more work needs to be done to represent terminology and value sets and to model future temporal action in CDS recommendations.

## Abbreviations

AIR: Acute Inflammatory Response
CCSR: Canadian Cervical Spine Rule
CDS: clinical decision support
CELS: Clinical Evidence Logic Statement
CQL: Clinical Quality Language
CT: computed tomography
ELM: Expression Logical Model
FHIR: Fast Healthcare Interoperability Resources
HL7: Health Level Seven
HLE: Harvard Medical School Library of Evidence
IT: information technology
JSON: JavaScript Object Notation
MRA: magnetic resonance angiography
MRI: magnetic resonance imaging
NEXUS: National Emergency X-Radiography Utilization Study
OPTIMAP: optimizing imaging in suspected appendicitis
PAMA: Protecting Access to Medicare Act
PE: pulmonary embolism
QUICK: Quality Information and Clinical Knowledge
rGeneva: Revised Geneva
SCORE: simple calculated osteoporosis risk estimation
SMS: Simplified Motor Score
SNOMED CT: Systematized Nomenclature of Medicine—Clinical Terms
STONE: sex, timing, origin, nausea, erythrocytes
TOF: time-of-flight
VQ SPECT: ventilation-perfusion single-photon emission computed tomography
XML: Extensible Markup Language
